# TIA and minor stroke: a qualitative study of long-term impact and experiences of follow-up care

**DOI:** 10.1186/s12875-019-1057-x

**Published:** 2019-12-17

**Authors:** Grace M. Turner, Christel McMullan, Lou Atkins, Robbie Foy, Jonathan Mant, Melanie Calvert

**Affiliations:** 1 Institute of Applied Health Research, University of Birmingham, Birmingham, B15 2TT UK; 2 Centre for Patient Reported Outcomes Research, University of Birmingham, Birmingham, B15 2TT UK; 3 Centre for Behaviour Change, University College London, London, WC1E 6BT UK; 4 Faculty of Medicine and Health, University of Leeds, Leeds, LS2 9JT UK; 5 Department of Public Health and Primary Care, University of Cambridge, Cambridge, CB1 8RN UK

**Keywords:** Transient ischemic attack, Minor stroke, Quality of life, Rehabilitation, Qualitative

## Abstract

**Background:**

Transient ischaemic attack (TIA) and minor stroke are often considered transient events; however, many patients experience residual problems and reduced quality of life. Current follow-up healthcare focuses on stroke prevention and care for other long-term problems is not routinely provided.

We aimed to explore patient and healthcare provider (HCP) experiences of residual problems post-TIA/minor stroke, the impact of TIA/minor stroke on patients’ lives, and current follow-up care and sources of support.

**Methods:**

This qualitative study recruited participants from three TIA clinics, seven general practices and one community care trust in the West Midlands, England. Semi-structured interviews were conducted with 12 TIA/minor stroke patients and 24 HCPs from primary, secondary and community care. Data was analysed using framework analysis.

**Results:**

A diverse range of residual problems were reported post-TIA/minor stroke, including psychological, cognitive and physical impairments. Consultants and general practitioners generally lacked awareness of these long-term problems; however, there was better recognition among nurses and allied HCPs. Residual problems significantly affected patients’ lives, including return to work, social activities, and relationships with family and friends. Follow-up care was variable and medically focused. While HCPs prioritised medical investigations and stroke prevention medication, patients emphasised the importance of understanding their diagnosis, individualised support regarding stroke risk, and addressing residual problems.

**Conclusion:**

HCPs could better communicate information about TIA/minor stroke diagnosis and secondary stroke prevention using lay language, and improve their identification of and response to important residual impairments affecting patients.

## Background

Transient ischaemic attack (TIA) and minor stroke are important risk factors for stroke. Over 46,000 people experience a first TIA/minor stroke per year and 510,000 people live with TIA/minor stroke in the United Kingdom [1]. National guidelines promote rapid diagnosis and long-term management that focuses on stroke prevention [2–4]. A growing body of epidemiological research demonstrates that many people with TIA/minor stroke experience residual impairments, [5–8] reduced quality of life [9–11] and difficulty returning to work or usual activities [12–15].

Qualitative studies of TIA/minor stroke have predominantly focussed on patients’ experiences of initial symptoms, symptom recognition and help seeking behaviour [16–19]. However, some studies have explored patients’ experiences after the acute stage and reported a diverse range of residual impairments, including: anxiety, [20–26] mood/emotional impact, [20, 21, 23, 26] cognitive impairment, [21, 26] fatigue, [23, 25, 27] physical weakness, [21, 23, 25] visual impairments [25] and impaired speech [21]. The impact of TIA/minor stroke on patients’ ability to return to work, [25–27] performance at work, [20, 25] social activities [20, 21, 23, 26, 27] and family relationships has also been reported [23–26, 28]. However, these studies only focus on TIA/minor stroke patients’ experiences and there is no qualitative exploration of healthcare providers’ (HCPs) perspectives. To improve future care, it is important to identify any critical gaps in understanding and experiences of TIA/minor stroke between HCPs and patients.

This qualitative study aimed to explore patient and HCP experiences of: (i) residual problems post-TIA/minor stroke; (ii) the impact of TIA/minor stroke on patients’ lives; and (iii) current follow-up care and sources of support.

## Methods

This study is a qualitative study with TIA/minor stroke patients and HCPs. Qualitative methodology was used as it is best placed to describe participants’ views and experiences of disease, and impact of disease and related healthcare [29].

This study is nested within a larger research programme aiming to develop and assess the feasibility of a follow-up pathway post-TIA/minor stroke (SUPPORT TIA: Structured follow-Up Pathway to imProve management Of Residual impairmenTs and patients’ quality of life after Transient Ischaemic Attack and minor stroke) [30]. The qualitative findings will inform design of an intervention follow-up pathway for TIA/minor stroke patients.

### Participants, sampling and recruitment

Participants were: (i) people who have experienced a TIA or minor stroke or (ii) HCPs working with TIA/minor stroke patients, including: secondary care doctors, nurses or allied health professionals (AHPs); general practitioners (GPs); and community AHPs and nurses.

Eligibility criteria for TIA/minor stroke patients were: confirmed diagnosis by a stroke consultant or clinical code in primary care medical records verified by a GP (TIA); modified Rankin scale score ≤ 1 (minor stroke) [31]; aged ≥18 years; ability to converse in everyday English; capacity to provide fully informed consent; no history of stroke or dementia; and no reasons known to the clinician to exclude (e.g. terminal illness, recent bereavement). Eligible HCPs were current members of staff from secondary, primary or community care who worked with TIA/minor stroke patients.

Convenience and snowball sampling was used initially; however, sampling became increasingly purposeful to achieve variation in diagnosis and time since event for patients, and clinical role (doctor, nurse, AHP) for HCPs. TIA/minor stroke patients were recruited from two general practices and TIA clinics at three hospitals in the West Midlands, England. Postal invitation was used to recruit patients from the general practices and patients from TIA clinics were invited to participate by a member of the clinical team. Secondary care doctors, nurses and AHPs were recruited from the three TIA clinics. Community HCPs were recruited from Birmingham Community Healthcare Trust. GPs were recruited from two general practices in the West Midlands.

### Data collection

One-to-one, semi-structured interviews were conducted by telephone or face-to-face (at the University of Birmingham, participants’ home, or participants’ workplace). No repeat interviews were conducted. All interviews were conducted by GT, a female, non-medical researcher trained in qualitative research methods. The interviewer did not have a relationship with any of the participants; however, a small number of HCPs (*n* = 2) were known contacts.

Topic guides were informed by existing literature, consultation with the research team and patient partners, and refined through piloting. Topic guides covered: residual problems post-TIA/minor stroke; impact on patients’ lives; follow-up care; and sources of support (see Additional file 1).

Participants completed a short demographic questionnaire (see Additional files 2 and 3) and field notes were taken during interviews. Digital audio recorded interviews were transcribed verbatim by a professional transcription service. Transcripts were not returned to participants for feedback.

Interviews were conducted between March and November 2018, until the research team judged that the sample and data had sufficient depth and breadth to address the research questions.

### Data analysis

Computer Aided Qualitative Data Analysis Software QSR NVivo 12 supported the sorting, coding and organisation of transcribed data prior to application of the framework method [32]. Transcripts were read several times to enable familiarisation with the interviews. GT coded all transcripts and CM (experienced qualitative researcher) independently coded a subset (10%). Open coding was initially applied to three transcripts independently by GM and CM, codes were then reviewed by GM and CM and discrepancies resolved through discussion. Agreed codes were then organised into categories which formed the analytical framework. This framework was applied to all transcripts and iteratively refined. Microsoft Excel was used to generate a matrix and data were ‘charted’ into the matrix by GT. The matrix was used to describe participants’ experiences and facilitate comparisons within and across participant groups. The final analysis and interpretation was discussed with the wider research team, including patient partners.

### Ethics, consent and permissions

Favourable ethical opinion was given by the Warwickshire North West - Greater Manchester East Research Ethics Committee (Reference: 17/NW/0737). Written or recorded verbal consent was obtained from the participants, by the interviewer, immediately prior to the interview.

## Results

The final sample consisted of 12 patients (7 TIA and 5 minor stroke) and 24 HCPs (5 stroke doctors, 4 nurses, 9 AHPs and 6 GPs). Participants’ characteristics are summarised in Tables 1 and 2 and detailed in (see Additional file 4: Tables S1 and S2). The mean interview length was 49 min (range 23 to 89 min).

**Table 1:** Characteristics of TIA and minor stroke participants (*n* = 12)

Variable	Number (%)
Diagnosis	
TIA	7 (58.3)
Minor stroke	5 (41.7)
Number of TIAs/ minor strokes	
1	7 (58.3)
2	4 (33.3)
3	1 (8.3)
Time since latest TIA/ minor stroke	
< 6 months	5 (41.7)
6–12 months	2 (16.7)
13–24 months	3 (25.0)
> 24 months	2 (16.7)
Age	
< 45 years	2 (16.7)
46–60 years	6 (50.0)
> 60 years	4 (33.3)
Sex	
Male	4 (33.3)
Female	8 (66.7)
Ethnicity	
White	11 (91.7)
Asian	1 (8.3)
Employment	
Employed- full time	5 (41.7)
Employed- part time	0 (0.0)
Unemployed	2 (16.7)
Student	1 (8.3)
Retired	3 (25.0)
Semi-retired	1 (8.3)

**Table 2:** Characteristics of healthcare provider participants (*n* = 24)

Variable	Number (%)
Age (years)	
21–30	1 (4.2)
31–40	8 (33.3)
41–50	12 (50.0)
51–60	3 (12.5)
Sex	
Male	10 (41.7)
Female	14 (58.3)
Profession	
Stroke consultant	5 (20.8)
Nurse	4 (16.7)
Allied health professional	9 (37.5)
GP	6 (25.0)
Healthcare setting	
Secondary care	9 (37.5)
Primary care	6 (25.0)
Community care	6 (25.0)
Secondary & community care	3 (12.5)
Years of experience	
< 5	3 (12.5)
5–10	5 (20.8)
11–20	11 (45.8)
> 20	5 (20.8)

**Fig. 1:**
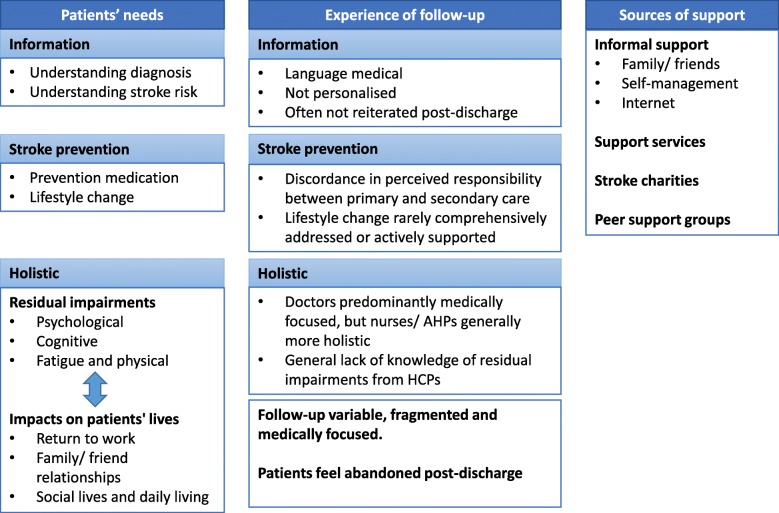
Summary of results

Results are described below, grouped according to research questions (Fig. 1).

### Residual problems

Patients and HCPs reported a diverse range of residual problems post-TIA/minor stroke, including psychological, cognitive and physical impairments (see Additional file 4: Tables S3). Although all participant groups mentioned anxiety and fatigue, patients emphasised these as the most significant sequelae. Some patients described residual problems as “hidden”, due to lack of physical symptoms, and consequently found their symptoms were dismissed by HCPs or felt they weren’t “entitled” to seek help for non-physical problems.

“*I know it’s there but people who look at me don’t see it. They say oh you look okay. That’s the worst thing is when you say, oh you look okay, I might look good, I don’t feel it you know*” [P1, TIA].

Nurses and AHPs were usually aware of residual impairments post-TIA/minor stroke. However, these problems were under-recognised by other HCPs, particularly consultants, who predominantly saw patients at the acute stage, and GPs, who infrequently saw TIA/minor stroke patients.

“*It’s not a recognised phenomenon but we know it is and we see it all the time. So, fatigue, anxiety and loss of confidence and fear. They would be the four main ones for me that we would see.*” [H22, stroke nurse].

### Psychological problems

Most participants described psychological consequences of TIA/minor stroke, particularly anxiety about having a full stroke. Depression was reported, but less frequently, and was considered by some HCPs to be a consequence of other residual problems, such as anxiety or fatigue which can “*snowball into depression*” [H9, GP], or an exacerbation of pre-stroke psychological problems. For instance, one patient became severely depressed and suicidal after cognitive impairment post-TIA affected their ability to work in their cognitively demanding job. Mood and emotional problems described included: increased emotionalism, anger, mood swings, frustration, irritation, lack of empathy and loss of confidence. Sometimes these were in reaction to residual symptoms (such as fatigue) or impacts on life (such as inability to work).

### Cognitive impairment

Patients, AHPs and GPs all reported cognitive impairments, which were subtle and covered four domains: executive functioning, memory, attention and language. Some cognitive problems were difficult to articulate:

“M*y head don’t feel right every day, all day, there’s something not right with it, I couldn’t put my finger on it*” [P1, TIA].

Executive functioning problems included difficulties following instructions, preparing meals and planning journeys. Four patients reported memory problems; two were subtle but two had significant impacts on lives. Attention deficits included problems maintaining concentration and difficulty following group conversations. One patient had cognitive-related speech problems, particularly finding words, which caused frustration and significantly affected confidence.

AHPs highlighted impacts of “hidden” cognitive problems on patients’ lives, such as return to work or ability to self-assess driving competence, and recognised level of impact was context specific so tailored screening tests and treatment accordingly.

### Fatigue and physical problems

Fatigue was frequently reported and affected patients’ daily lives, ability to work, performance at work, confidence and mood. Fatigue was particularly debilitating for patients with work and family commitments.

*“[fatigue] that’s the other biggest impact because I literally, I can’t do too much …*” [P7, TIA].

Some participants highlighted that fatigue was most severe and debilitating in the first 2 to 4 weeks post-TIA/minor stroke. At this time patients reported sleeping all day, being unable to work and relying on family for child care. For some patients, fatigue resolved after a month; however, six patients reported persistent fatigue and tried different coping strategies, including power naps, regulating sleep, resting after work and exercise. Nurses and AHPs suggested reassurance that fatigue is normal often helped patients; however, in some cases a more proactive approach was required, such as education, sleep hygiene (sleep behaviours and habits) and adjusting daily routines.

Physical symptoms were less common, those reported included: minor weakness, “altered sensation”, pain, speech, headaches and problems swallowing. Most physical problems were considered minor by patients with very limited impact on their lives; however, speech impairment or pain significantly affected quality of life for three patients.

“*… I have pain in my face and my ear and my hand … when there’s too much input, it gets too much in my head and then the pain comes*.” [P9, minor stroke].

In contrast, two patients did not experience any residual problems and felt they had returned to normal. HCPs also reported some patients had no issues and were happy to “move on”.

### Impact on patients’ lives

The impact of TIA/minor stroke reported by patients and HCPs was diverse, including: return to work/education; relationships with family/friends; social lives; and daily activities (see Additional file 4: Table S4). Many patients experienced loss of identify and some struggled to accept their diagnosis, particularly patients < 65 years or who considered themselves healthy.

### Return to work or education

Eight patients were working at the time of their TIA/minor stroke and one was a mature student (a person who begins their studies at university or college a number of years after leaving school). All took time off work/education, varying from one to 3 months, and most had a phased return. Some HCPs recognised the impact of residual problems on ability to work, particularly for patients in cognitively demanding jobs; however, others were sceptical about impact of TIA/minor stroke on ability to work and one consultant felt some patients used TIA as an excuse to stop working.

Patients’ reported ability to work was affected by cognitive problems (particularly memory or concentration), fatigue, anxiety, loss of confidence, feeling overwhelmed and driving restrictions.

“*I need to take more breaks, I cannot sit in two, three hour meetings, that kills me so I need to have more breaks and I need to write much more things down, I’m still doing things forgetting that I’ve done it or forgetting that I haven’t done it, I put in the same meeting twice with the team … I’m almost up to full time … but not full capacity because I notice that I’m not this quick or smart as … , I need to focus much more*.” [P9, minor stroke].

Impacts on work reported by patients included: reduced performance at work; decreased workload/hours; giving up opportunity for promotion (due to lost confidence); and changing jobs to improve lifestyle. One patient, who had a cognitively demanding role, lost his job due to mild cognitive problems which resulted in depression, suicidal feelings and financial problems. Other patients experienced a loss of identity and helplessness about the impact of residual impairments on their ability to work.

### Relationships with family/ friends

Impacts on relationships with family/friends, both negative and positive, were discussed by half the patients but only three HCPs (a GP and two AHPs). Relationships were negatively affected by patients’ mood and emotional problems; patients becoming withdrawn due to difficulty engaging in group conversations; and family/friends disregarding residual problems.

“*Mood swings are the worst for my wife, she suffers the most … so I try to watch what I say, most times it’s quietness between us because I’m scared to say something that will just trigger one of these* [mood swing] *it’s a stressful time for her …*” [P1, TIA].

HCPs reported changes in family roles and dynamics with other family members having to take over household responsibilities (such as childcare or finances) and family members’ fear of the patient having a full stroke, which were corroborated by patients.

In contrast, some patients reported positive impacts, such as improved family relationships and re-evaluation of work-life balance or family priorities.

### Social lives and daily living

Many patients described negative impacts on social lives, such as residual problems or loss of confidence preventing participation in social activities, hobbies, exercise or group conversations. Some patients described the loss from being unable to participate in their usual activities.

“*My quality of life is affected because I can’t go back to doing things that I like to do, I haven’t got … I’m not working at the moment, I do piano lessons, I’m not doing piano lessons, I’m not going to the gym to the same degree, my personal trainer basically dropped me because I couldn’t really do very much so you know it’s like all of that that you have lost …*” [P7, TIA].

In contrast, HCPs did not mention social lives and often underestimated impact of TIA/minors stroke on patients’ lives. However, some HCPs acknowledged negative impacts on daily living, including looking after children/grandchildren, managing household finances and insurance; were aware that driving restrictions affected daily routines, such as picking up children from school; and recognised challenges of lifestyle changes. For instance, one AHP described a patient who gave up cigars and alcohol easily but when told not to drink coffee “… *he nearly collapsed, cause that’s the last little thing … it might feel small to us but that was his last pleasure*” [P15, psychologist].

### Experience of follow-up care and sources of support

Follow-up care varied in terms of organisational structures and practices of individual HCPs. For example, only one of the three hospitals offered nurse-led follow-up, which was inconsistently used by consultants. Patients reported mixed experiences of follow-up care, but largely felt abandoned and alone post-discharge. Patients’ needs broadly comprised: information, stroke prevention and holistic care (see Additional file 4: Table S5).

### Information needs

Information regarding diagnosis and stroke risk was predominantly provided at the acute stage and considered inadequate by most patients. Patients reported information was difficult to process at the time of their diagnosis; language was too medical; HCPs gave contradictory advice; and information was too generic and not personalised. HCPs were generally aware that many patients lacked basic understanding about their diagnosis and stroke risk/prevention; this was corroborated in some patient interviews:

[explaining why they did not seek medical care for subsequent TIA symptoms] “*Well it [TIA symptoms] only lasted you know 20 minutes … So the next one will probably only last a minute, so I didn’t bother.*” [P4, TIA].

Secondary care clinicians recognised it is not ideal to deliver information at time of diagnosis and information should be reiterated by primary/community HCPs. However, most GPs admitted not repeating information or checking patients’ understanding due to time constraints or assumptions this had been done adequately in secondary care.

Most patients accessed information online, but often found this overwhelming, confusing, contradictory and too generalised. In contrast, some patients found stroke websites useful and learnt from other patients’ experiences from forums. Consultants, nurses and AHPs frequently relied on stroke charity websites to supplement verbal information. Patients often relied on family/friends to explain medical terms, search for information online and help with treatment decisions.

### Stroke prevention

There were conflicting views between primary and secondary care clinicians regarding responsibility for prescribing prevention medication. Some consultants felt it was their role and followed up patients to monitor progress. Others had a “protocolised” approach (prescribed the same medication regardless of the patient) or relied on GPs to prescribe appropriate medication. Some GPs considered they were best placed to prescribe prevention medication with their knowledge of patients’ comorbidities and polypharmacy. However, other GPs felt they lacked specialist stroke knowledge, particularly when first line drugs were contraindicated, or time/resource constraints prevented them checking hospital prescriptions or patients understanding.

“*Rightly or wrongly I think we have to really make the assumption that the patient has been counselled adequately about that medication and why they’re being put on it … it wouldn’t be feasible for every specialist letter we get for strokes and everything else to contact the patient to sort of go through the, we wouldn’t do anything else really. So we add the medication to the repeat prescription …*” [GP, H13].

Being prescribed lifelong medication was a significant change for patients and many felt unsupported. Nurses recalled some patients misunderstanding prevention medication, such as thinking it was short-term.

Lifestyle change was not comprehensively addressed by HCPs, usually because of time restraints. Some HCPs mentioned healthy lifestyle, but did not actively support patients to make changes. The only exceptions were AHPs who saw this as part of their role.

“*So, we talk about stopping smoking and healthy diet and exercise but it’s a fairly brief discussion and don’t really feel I have time in the clinic to do that in great depth*.” [consultant, H20].

### Holistic needs

Follow-up care, particularly from consultants and GPs, was predominantly medically focused. Some consultants felt they lacked skills to address holistic needs and there was a general lack of knowledge among consultants and GPs of residual problems post-TIA/minor stroke.

“*I don’t think I will bring back somebody to manage their mood and fatigue because I don’t feel competent in doing that and probably I’m not*.” [H17, consultant].

Nurses and AHPs generally provided more holistic care; however, most patients in our sample did not access nurse/AHP follow-up. Some HCPs felt patients were more likely to talk to nurses about holistic needs than doctors. This was corroborated by some patients who considered doctor appointments were only for medical issues.

Patients often relied on informal sources of support for holistic needs. Family/friends provided emotional and practical support (such as household responsibilities and childcare). Some patients employed self-management strategies, particularly for fatigue (regulating sleep patterns, exercise, naps and planning activities); cognition (word searches, crosswords and jigsaws); and anxiety (mindfulness and relaxation techniques).

Some patients accessed support services. Three patients received psychological support through their GP, work or self-referral after signposting from a stroke charity. Two minor stroke patients had therapist support through the hospital or their workplace. HCPs occasionally referred patients to support services but generally lacked awareness of what is available.

HCPs considered the Stroke Association (UK’s biggest stroke charity) a valuable resource for additional support when they had limited time or lacked expertise to address holistic factors. However, patients varied in their perception of and engagement with this charity. Some patients felt stroke charities were only for people with full stroke, therefore, did not deserve their support despite experiencing significant residual problems. Others received useful support from stroke charities, including advice and signposting to community services. Similarly, patients had mixed responses to stroke support groups. Most patients stated they would feel “embarrassed” or like a “fraud” attending groups with full stroke patients, despite wanting peer support; whereas, others benefitted from such groups.

## Discussion

### Summary

This is the first qualitative study to explore patient and HCP experiences of long-term impacts of TIA/minor stroke and follow-up care. Many patients experienced a diverse range residual problems, including psychological, cognitive and physical impairments, which were ‘hidden’ but had impacts on their lives. Stroke consultants and GPs generally lacked awareness about residual problems post-TIA/minor stroke; however, there was better recognition among nurses and AHPs. Follow-up care was variable, medically focused and did not adequately address patients’ complex, individual needs. While HCPs prioritised medical investigations and stroke prevention medication, patients concerns encompassed understanding their diagnosis, individualised support to manage their stroke risk, and addressing impacts of residual problems. Many patients felt abandoned post-discharge and relied on support from family/friends, the internet and self-management strategies.

### Strengths and limitations

We drew upon a wide range of experiences, including patients (at a range of time points post-event and age ranges) and HCPs across different healthcare settings and disciplines. These different perspectives have enriched our understanding of the diverse range of symptoms/impacts post-TIA/minor stroke and disparate priorities for follow-up care between patients and HCPs. Despite recruitment from ethnically diverse populations in the West Midlands, most participants were white. For pragmatic reasons, only a subset of transcripts were double coded.

### Comparison with existing literature

Our results support findings from other studies which report residual impairments post-TIA/minor stroke [20–27]. Similar to our study, TIA/minor stroke has been demonstrated to impact on patients’ ability to return to work or usual activities, and can affect relationships with family and friends [20, 21, 23, 25–28]. In addition, the ‘hidden’ nature of impairments post-TIA/minor stroke has been reported to cause difficulties for patients to communicate their needs [26] and frustration due to lack of recognition of problems from HCPs and family/friends [20]. Our study is the first to report HCPs perspectives of residual problems post-TIA/minor stroke and highlight the general lack of awareness about these problems, particularly from consultants and GPs.

Few studies have explored TIA/minor stroke patients’ experience of follow-up care or sources of support. Those that did focused on stroke prevention and none have explored HCPs’ perspectives. Hillsdon et al. (2013) found patients were unable to digest information delivered at the acute stage and used other sources, such as internet and peers, for information about diagnosis and stroke prevention [28]. Other studies found patients did not receive formal support for secondary stroke prevention and patients’ actions were self-directed, driven by personality traits (e.g. optimistic or competitive personalities) [26] and correlated with perceptions of future stroke risk [22]. Similar to our findings, other studies have reported patients’ dissatisfaction with care, particularly lack of: communication, [28] holistic follow-up, [25] rehabilitation options, [26] and individualised information and support [28]. Our findings further enrich understanding of the variability in follow-up care, complexity of patients’ needs and differences in priorities for follow-up care between patients and HCPs.

### Implications for research and/or practice

Current healthcare post-TIA/minor stroke is variable and does not address patients’ complex, individual needs. Our findings suggest follow-up needs to encompasses information provision (diagnosis and stroke risk); stroke prevention (medication and lifestyle change); and holistic needs (residual problems and return to work/usual activities).

Adequate information provision requires lay language, two-way communication and information reiterated at multiple time points by different HCPs. Lifestyle change could be actively addressed by HCPs with consideration of the patients’ individual context and support from community support services. HCPs need to consider the diverse range of residual symptoms and problems TIA/minor stroke patients may experience. Proactive identification of such issues by HCPs might be beneficial.

Greater integration between secondary and primary care clinicians and better distinction of their roles might improve stroke prevention by combining specialists’ stroke knowledge with generalists’ knowledge of the patient. Tailored support for patients could potentially be further improved by better access to AHPs and nurses, and continuous communication loops with primary and secondary care physicians. Due to rapid discharge of patients from secondary care, primary care clinicians in particular have an important role in the coordination and communication of tailored support between healthcare settings and managing care in consideration with multimorbidity, polypharmacy and patients’ preferences.

Future research is required to establish the natural history of residual impairments post-TIA/minor stroke, including onset and duration, to help inform optimal time points for follow-up. Understanding the mechanism and characteristics of residual problems would help inform appropriate management/treatment approaches. For example, Chun et al. (2018) found anxiety post-stroke/TIA is predominantly phobic [33]. In addition, our findings suggest the value of collecting data on residual symptoms and impacts on patients’ lives in future TIA/minor stroke research studies. Further research should evaluate structured models of care to improve follow-up post-TIA/minor stroke. There is a need to develop pathways which are more responsive to TIA/minor stroke patients’ needs and are supported by education to ensure HCPs are sensitive to these needs and have skills to address them.

## Conclusion

Our findings demonstrate the complexity and variability of TIA/minor stroke patients’ needs and impacts on their lives. Optimal follow-up might address information provision, secondary stroke prevention and holistic needs. However, current healthcare post-TIA/minor stroke is limited and predominantly medically focused whist HCPs can miss opportunities to respond to patients concerns that have significant implications for quality of life and stroke risk.

## Supplementary information

**Additional file 1.** Topic guides. Topic guides for patients and healthcare providers

**Additional file 2.** Demographic questionnaire. Demographic questionnaire for patient participants

**Additional file 3.** Demographic questionnaire Demographic questionnaire for healthcare provider participants

**Additional file 4: Table S1.** Characteristics of TIA and minor stroke participants. **Table S2.** Characteristics of healthcare provider participants. **Table S3.** Residual impairments post-TIA and minor stroke: subthemes and example quotes. **Table S4.** Impact of TIA and minor stroke on patients’ lives: subthemes and example quotes. **Table S5.** Experience of follow-up care and sources of support post-TIA and minor stroke: subthemes and example quotes

## Data Availability

The datasets used and/or analysed during the current study are available from the corresponding author on reasonable request.

## Abbreviations

AHP: Allied Health Professionals
GPs: General Practitioners
HCP: Healthcare Provider
TIA: Transient Ischaemic attack
