# Supplementary material for: TIA and minor stroke: a qualitative study of long-term impact and experiences of follow-up care
Source: BMC Fam Pract. 2019 Dec 17;20:176. doi: 10.1186/s12875-019-1057-x (PMC6918619; doi:10.1186/s12875-019-1057-x)
Supplement: Supplementary file 1 — Additional file 1. Topic guides. Topic guides for patients and healthcare providers [file 12875_2019_1057_MOESM1_ESM.docx]

Topic guides

# TIA/ minor stroke patients

***In the first part of the interview I’m going to ask questions about what impact, if any, your mini stroke has had on your life and your experience of healthcare after your mini stroke. Then in the second part of the interview I will ask your opinion on a new follow-up pathway we are designing.***

## Impact of TIA/ minor stroke and experience of healthcare

**Can you tell me about your experience of having a TIA?**

- First TIA?
- Other comorbidities?
- Were you working?

**What has changed since your TIA?**

**How has it affected you emotionally?**

**Has there been any impact on your memory or cognition?**

**Impact on energy levels?**

**Do you have any physical effects, like weakness?**

**Do you feel the TIA has had any impact on your relationship with your family/ friends?**

**What about your social life?**

- - Is there anything you have not been able to do since the TIA?
  - Has it had any impact on activities you enjoy?

**[If working] Could you tell me what impact, of any, your TIA had on your work?**

- Did you take any time off work?
- Do you have any difficulties at work?
- Have you made any changes to your work?
- Did you get any help returning to work?

**Any other impact?**

- Some people experience a change in… [energy levels, mood, memory or other cognition]

**Do you feel like you returned back to normal, how you were before?**

**What care did you receive in hospital?**

**Where did you get help or support from after you left hospital?**

- Did you see your GP/ go back to hospital/ see anyone from the community/ charities?
- Who arranged that?
- **Medical:** medication, stroke prevention: diet and lifestyle
- **Therapeutic**: anxiety, depression, fatigue, cognition
- **Social:** finance

**What do you think about the care you received?**

- In hospital
- From your GP

**Have you accessed any support service or resources?**

- How did you know what was available?

**Did you get any support from anyone else?**

- Family/ friends, charities, support groups, social media
- Would you mind telling me more about that?

**What were/ are your main concerns or questions after the TIA?**

**What information were you given about:**

- What caused your stroke
- How to prevent strokes in the future/ advice about diet/ exercise
- Potential impact e.g. fatigue

**Did you look for any information by yourself?**

**What did you find the most useful from your follow-up care?**

**What support do you feel like you need now?**

- Who could best provide this support?

## Opinions on how healthcare/ support can be improved

**How do you think healthcare/ follow-up could be improved for people who have had a TIA?**

- **Medical:** medication, stroke prevention: diet and lifestyle
- **Therapeutic**: anxiety, depression, fatigue, cognition
- **Social:** finance

**What do you think were your main needs after your TIA?**

**Would you prefer to go for follow-up care, like your GP practice or back to the hospital?**

- Why?

**Who would you want to be followed up by, like your GP, the stroke consultant, a nurse?**

- What about someone not clinical like a Stroke Association worker?

**We would are thinking of developing a new follow-up appointment at the hospital with a stroke nurse, would that have been useful for you?**

- What would have been the best time after your TIA?
- What would you want to get out of a follow-up appointment?

**Would you have been happy going back to the hospital to see a stroke nurse?**

- How do you feel about going back to the hospital
- Is there anything that would put you off going or any reasons you wouldn’t want to go?

**When is the best time to give people information about stroke and stroke prevention?**

- What is the best way to provide this information? Leaflets, websites?

**We are considering developing a directory of local support services or resources to help people find out what is available. Would that be something you would want to use?**

- What would be the best format? E.g. online
- What support services or resources would you be interested in knowing about?

**Do you have any other suggestions about how care after TIA could be improve or about what the care needs are?**

**Do you have any other comments or anything you want or revisit from before?**

**That’s all the questions I have, do you have any questions for me?**

# Secondary care

***In the first part of the interview I will ask you about what happens in your current practice and the second half of the interview will focus on your perspective on how to improve follow-up.***

## Current practice

**To start off, what is your role in treating people who have had a TIA or minor stroke?**

**What is the current healthcare pathway for TIA and minor stroke patients?**

**Can you tell me about the follow-up for TIA/ minor stroke patients?**

- How many patients attend this follow-up appointment?
- What information do you give people at their follow-up appointment?
  - What format e.g. leaflets?
- What do patients want to talk about at their follow-up?

**How do you identify the needs of TIA/ minor stroke patients at follow-up?**

- Do you use screening tools or questionnaires, like HADS/ MoCA?

**Do you ask patients about mood/ cognition/ fatigue/ social?**

- Do you see this as part of your role?
- Are you aware that some TIA/ minor stroke patients may have residual impairments like mild cognitive impairment or mood problems?
  - Have you ever encountered these types of problems with any of your TIA/ minor stroke patients?
    - Can you give an example? What did you do in this scenario?

**What would you normally do if you identified that someone had problems with mood, cognition or social problems?**

- Would you ever refer patients to support services?
  - How do you know or hear about what services are available?
  - How do you decide which support service to refer people to?

**How do you communicate patients’ needs with other healthcare settings, like primary/ community care?**

- What information do you put in the discharge letter?
- How could this be improved?

## Opinions on a structured follow-up pathway for TIA and minor stroke patients

***Now we’re going to talk about improving the follow-up pathway for TIA/minor stroke patients.***

**How do you think healthcare/ follow-up for TIA and minor stroke patients could be improved?**

***We want to improve holistic care for TIA and minor stroke patients which includes stoke education, prevention (both medical and lifestyle/diet) and potential long term residual impact, like fatigue, mood or cognitive problems.***

***We’re interested in developing a standardised follow-up pathway for TIA and minor stroke patients where all patients will get a nurse-led follow-up appointment at a TIA clinic at 4-6 weeks. The goal of this follow-up would be to identify patients’ needs, refer them to appropriate support services and provide information about their TIA, such as stroke risk and prevention.***

**What do you think needs to happen to make this pathway successful?**

Do you think you have good knowledge of what the potential needs of the patients are in terms of potential long-term sequelae, stroke prevention and education needs?

Would you feel confident in identifying people’s needs?

How about managing needs related to stroke prevention and education?

How about managing needs related to long-term sequelae, like fatigue, mood or cognition?

- Do you have good knowledge of what support services are available?

How do you decide what the best action plan is to address people’s needs?

To what extent do you see this as part of your role?

Do you have the necessary resources to be able to identify and address patient’s needs?

Do you think that action plans agreed at a follow-up appointment would get actioned, whether that’s getting the patient to self-refer or see their GP or patients being able to access recommended support services?

Barriers to communication with primary care?

Are there any other influences that could be barriers or facilitators to successful follow-up?

**What are the main barriers to being able to provide holistic care at a follow-up appointment?**

**Is there anything you think we are missing from the follow-up pathway?**

**Do you have any other comments/ is there anything we’ve discussed that you’d like to go back to?**

# Primary care

***In the first part of the interview I will ask you about what happens in your current practice and the second half of the interview will focus on your perspective on how to improve follow-up for TIA and minor stroke patients.***

## Current practice

**I’m interested in what happens to TIA patients after they’ve been discharged from clinic. After the acute stage, so post-diagnosis, in what context would you see TIA patients?**

- How often do you see people who have had a TIA or minor stroke in your GP practice?

**How do you know if one of your patients has had a TIA/ stroke?**

**How useful are the discharge letters?**

- **How could they be improved?**

**Do any of your patients have follow-up at TIA clinics?**

**What type of healthcare or support do you usually provide for TIA/ minor stroke patients?**

- Who initiates these appointments, i.e. you or the patients?
- What type of issues do you discuss?
- How often do you follow-patients up?

**How do you identify the needs of these patients?**

**Do you ask patients about mood/ cognition/ fatigue/ social?**

- Do you see this as part of your role?
- Do you use screening tools or questionnaires, like HADS/ MoCA?
- Are you aware that some TIA/ minor stroke patients may have residual impairments like mild cognitive impairment or mood problems?
  - Have you ever encountered these types of problems with any of your TIA/ minor stroke patients?
    - Can you give an example? What did you do in this scenario?
  - Do you ever inform patients that they might experience residual impairments?

**How would you normally treat someone that has problems with mood, cognition or social problems?**

**How do you normally identify or hear about support services for these impairments?**

- What about [community support/ internet sites/ peer support]
- How do you decide which support services to refer people to?

**How do you know about new guidelines or recommendations?**

## Opinions on a structured follow-up pathway for TIA and minor stroke patients

**How do you think healthcare/ follow-up for TIA and minor stroke patients could be improved?**

**We’re interested in developing a standardised follow-up pathway for TIA and minor stroke patients where all patients will get a nurse-led follow-up appointment at a TIA clinic. The goal of this follow-up would be to identify patients’ needs, refer them to appropriate support services and provide information about their TIA, such as stroke risk and prevention.**

**Primary care will have an important role in this pathway in managing the needs identified and longer term follow-up care.**

**What do you think needs to happen to make this pathway successful?**

- **Communication of needs from TIA clinics**
- **Act on recommendations**
- **Potential barriers**
  - Knowledge of long-term impact [will GPs recognise that TIA patients may experience long-term impacts?]
  - Knowledge of support services
  - Time constraints
  - Would you want to do your own assessment of the patients’ needs?
  - Proactive- asking patient to attend a follow-up appointment vs reliance on patient to make an appointment
  - What are the main barriers for TIA and minor stroke patients accessing support?

**What do you think would be the barriers to having the follow-up appointment in secondary care?**

**Is there anything you think we are missing or haven’t thought about?**

**Do you have any other comments or is there anything we’ve discussed that you’d like to go back to?**

***That is all the questions I have, is there anything you would like to ask? Thank you for your time.***

# Community healthcare

***In the first part of the interview I will ask you about what happens in your current practice and the second half of the interview will focus on your perspective on how to improve follow-up for TIA and minor stroke patients.***

## Current practice

**To start off, can you describe your role?**

- What type of patients do you normally see?
- Do you ever see people who have had a TIA or a minor stroke?

**What is the current healthcare pathway for your patients?**

**How do you identify the needs of your patients?**

- What about needs other than [physical rehabilitation/ speech therapy]?

**Do you ask patients about mood/ cognition/ fatigue/ social?**

- Do you see this as part of your role?
- Do you use screening tools (e.g. PH9)?
- Do you ever inform patients that they might experience these types of impairments?
- Are you aware that some TIA/ minor stroke patients may have residual impairments like mild cognitive impairment or mood problems?
  - Have you ever encountered these types of problems with any of your TIA/ minor stroke patients?
    - Can you give an example? What did you do in this scenario?

**What would you normally do if you identified that someone had problems with mood, cognition or social problems?**

**Do you ever talk to patients about preventing long-term problems, like anxiety or fatigue?**

**How do you normally identify or hear about support services for these impairments?**

- What about [internet sites/ peer support]
- How do you decide which support services to refer people to?

**In your opinion, what are the unmet needs for TIA and minor stroke patients?**

**What communication do you have with other healthcare settings, like primary/ secondary care?**

- How could this be improved?

## Opinions on a structured follow-up pathway for TIA and minor stroke patients

***Now we’re going to talk about improving the follow-up pathway for TIA/minor stroke patients.***

**How do you think healthcare/ follow-up for TIA and minor stroke patients could be improved?**

- What do you think a follow-up pathway for TIA and minor stroke patients should look like/ include?

**Where do you think is the best place to follow-up TIA/ minor stroke patients?**

- Why do you think that?
- Who do you think should follow up these patients?

**What would be the best time point after TIA/ minor stroke to have the follow-up appointment?**

- Why do you think that?
- How often do you think follow-up should happen?

**We want to improve follow-up for TIA and minor stroke patients. Our three key goals would be to: firstly be able to identify the needs of TIA and minor stroke patients; secondly to refer them to appropriate support services; and thirdly to provide information about their TIA/ minor stroke, such as stroke risk and prevention.**

**Thinking about the first goal of the pathway, what do you think is the best way to identify unmet needs of TIA and minor stroke patients?**

- What about screening tools?
- Would you feel confident in being able to identify the needs of TIA or minor stroke patients?
- What are the barriers to identifying patients’ needs?

**People will experience a spectrum of impairments after their TIA/minor stroke; for example, some people will have clinically diagnosable depression/ anxiety, whereas others will be sub-clinical but it still affects their quality of life. Which patients do you think our pathway should target?**

- Why do you think that?

**The second goal of the pathway is to refer people to appropriate support services. What are the main barriers for TIA and minor stroke patients accessing support?**

**We want to develop a directory of local support services to help people identity what support is available, how useful would this would be?**

- Do you think it would be useful for patients to access the directory? Why
- What do you think would be the best format for this directory; for example online?
- Would there be a better way to make people aware of support services?

**The third goal of the pathway is about information giving, what is the best way to provide people with information?**

- How confident would you feel in providing people with information about their TIA/ minor stroke and stroke prevention?
- What makes you feel that confident/ not confident?

**What do you think would be the barriers to having the follow-up pathway in community care?**

**Is there anything you think we are missing or haven’t thought about?**

**Do you have any other comments or is there anything we’ve discussed that you’d like to go back to?**

***That is all the questions I have, is there anything you would like to ask? Thank you for your time.***
