# Supplementary material for: TIA and minor stroke: a qualitative study of long-term impact and experiences of follow-up care
Source: BMC Fam Pract. 2019 Dec 17;20:176. doi: 10.1186/s12875-019-1057-x (PMC6918619; doi:10.1186/s12875-019-1057-x)
Supplement: Supplementary file 2 — Additional file 2. Demographic questionnaire. Demographic questionnaire for patient participants [file 12875_2019_1057_MOESM2_ESM.pdf]

UNIVERSITY OF BIRMINGHAM | CPROR CENTRE FOR PATIENT REPORTED OUTCOMES RESEARCH

NHS National Institute for Health Research

Support TIA

SUPPORT TIA: Structured follow-up pathway to improve ongoing impairments after TIA and minor stroke

Information about you

1. Are you:

☐ Male ☐ Female ☐ Prefer not to say

2. What is your age? __________ Years

3. Are you currently employed?

☐ Employed- Full time ☐ Unemployed

☐ Employed- Part time ☐ Retired

☐ Other, Please state ____

4. What is your ethnic group?

☐ White ☐ Mixed/ multiple ethic groups

☐ Asian/ Asian British ☐ Black/ African/ Caribbean/ Black British

☐ Other, Please state ____

7. How many mini strokes or minor strokes have you had? ______________

8. What were the approximate date(s) of your mini stroke(s) or minor stroke(s)?

____

____

Thank you for taking the time to complete this questionnaire
