# Supplementary material for: TIA and minor stroke: a qualitative study of long-term impact and experiences of follow-up care
Source: BMC Fam Pract. 2019 Dec 17;20:176. doi: 10.1186/s12875-019-1057-x (PMC6918619; doi:10.1186/s12875-019-1057-x)
Supplement: Supplementary file 3 — Additional file 3. Demographic questionnaire Demographic questionnaire for healthcare provider participants [file 12875_2019_1057_MOESM3_ESM.pdf]

UNIVERSITY OF BIRMINGHAM | CPROR CENTRE FOR PATIENT REPORTED OUTCOMES RESEARCH

NHS National Institute for Health Research

Support TIA

SUPPORT TIA: Structured follow-up pathway to improve ongoing impairments after TIA and minor stroke

Information about you

1. Are you:

☐ Male ☐ Female ☐ Prefer not to say

2. What is your age? __________ Years

3. Which healthcare sector do you work in?

☐ Primary care ☐ Secondary care

☐ Community care ☐ Third/ voluntary sector

☐ Other, Please state ____

4. Which of the following best describes you?

☐ Doctor ☐ Allied Health Professional

☐ Nurse ☐ Health worker

☐ Volunteer ☐ Other, Please state ___________________________

7. How many years of experience do you have as a healthcare provider/ volunteer?

______________________ Years

8. Do you have any experience of working with TIA or minor stroke patients?

☐ Yes ☐ No

Thank you for taking the time to complete this questionnaire
