# Supplementary material for: TIA and minor stroke: a qualitative study of long-term impact and experiences of follow-up care
Source: BMC Fam Pract. 2019 Dec 17;20:176. doi: 10.1186/s12875-019-1057-x (PMC6918619; doi:10.1186/s12875-019-1057-x)
Supplement: Supplementary file 4 — Additional file 4: Table S1. Characteristics of TIA and minor stroke participants. Table S2. Characteristics of healthcare provider participants. Table S3. Residual impairments post-TIA and minor stroke: subthemes and example quotes. Table S4. Impact of TIA and minor stroke on patients’ lives: subthemes and example quotes. Table S5. Experience of follow-up care and sources of support post-TIA and minor stroke: subthemes and example quotes [file 12875_2019_1057_MOESM4_ESM.docx]

# Supplementary tables

**eTable 1: Characteristics of TIA and minor stroke participants**

| **ID** | **Diagnosis** | **Number of TIAs/ minor strokes** | **Time since latest TIA/ minor stroke** | **Age (years)** | **Sex** | **Ethnicity** | **Employment** |
| --- | --- | --- | --- | --- | --- | --- | --- |
| P1 | TIA | 3 | 4 months | 51-60 | Male | White | Unemployed |
| P2 | TIA | 1 | 8 years | 61-70 | Female | White | Retired/ Volunteer |
| P3 | Minor stroke | 1 | 2 years | 41-50 | Male | White | Employed: part time |
| P4 | TIA | 2 | 2 years | 61-70 | Male | White | Unemployed |
| P5 | TIA | 1 | 11 years | 71-80 | Female | White | Retired |
| P6 | TIA | 2 | 2 years | 51-60 | Male | White | Semi-retired |
| P7 | TIA | 1 | 3 months | 51-60 | Female | White | Employed: full time |
| P8 | TIA | 1 | 3 months | 41-50 | Female | White | Employed: full time |
| P9 | Minor stroke | 2 | 1 year | 41-50 | Female | White | Employed: full time |
| P10 | Minor stroke | 2 | 11 months | 51-60 | Female | White | Student |
| P11 | Minor stroke | 1 | 4 months | 81-90 | Female | White | Retired |
| P12 | Minor stroke | 1 | 3 months | 51-60 | Female | Asian | Employed: full time |

**eTable 2: Characteristics of healthcare provider participants**

| **ID** | **Age (years)** | **Sex** | **Profession** | **Healthcare setting** | **Years of experience** |
| --- | --- | --- | --- | --- | --- |
| H1 | 21-30 | Female | AHP (Physio) | Community care | 5 |
| H2 | 41-50 | Female | AHP (SLT) | Community care | 3 |
| H3 | 41-50 | Female | AHP (Physio) | Secondary & community care | 23 |
| H4 | 51-60 | Female | Nurse | Community care | 37 |
| H5 | 41-50 | Female | AHP (OT) | Community care | 16 |
| H6 | 41-50 | Female | AHP (Physio) | Community care | 18 |
| H7 | 51-60 | Male | Stroke consultant | Secondary care | 20 |
| H8 | 31-40 | Male | GP | Primary care | 17 |
| H9 | 31-40 | Male | GP | Primary care | 6 |
| H10 | 51-60 | Male | GP | Primary care | 31 |
| H11 | 41-50 | Male | GP | Primary care | 18 |
| H12 | 31-40 | Male | GP | Primary care | 7 |
| H13 | 31-40 | Male | GP | Primary care | 13 |
| H14 | 31-40 | Female | AHP (OT) | Community care | 17 |
| H15 | 41-50 | Female | AHP (Psychologist) | Secondary & community care | 4 |
| H16 | 31-40 | Female | AHP (Physio) | Secondary & community care | 4 |
| H17 | 41-50 | Female | Stroke consultant | Secondary care | 8 |
| H18 | 41-50 | Female | AHP (Psychologist) | Secondary care | 12 |
| H19 | 41-50 | Female | Nurse | Secondary care | 12 |
| H20 | 41-50 | Male | Stroke consultant | Secondary care | 22 |
| H21 | 41-50 | Male | Stroke consultant | Secondary care | 24 |
| H22 | 31-40 | Female | Nurse | Secondary care | 13 |
| H23 | 31-40 | Female | Nurse | Secondary care | 10 |
| H24 | 41-50 | Male | Stroke consultant | Secondary care | 12 |
| AHP: Allied Health Professional; OT: Occupational Therapist; Physio: Physiotherapist; SLT: Speech and Language Therapist | | | | | |

**eTable 3: Residual impairments post-TIA and minor stroke: subthemes and example quotes.**

| **Subtheme** | **Quote** |
| --- | --- |
| **Psychological** |  |
| - Anxiety | “Then there is another bunch of patients who become very anxious … They think they’re going to have stroke at any minute and so they get very very anxious.” [H21, consultant]  “I mean I referred myself for counselling because I can’t cope with the anxiety, I don’t want to feel anxious all my life…” [P7, TIA] |
| - Depression | “I’m thinking it would be easier just to go [referring to suicide] and that’s the way I feel, it’s a terrible feeling, terrible thing to say but that’s the way I’ve been feeling lately, I think I’m struggling everything I do, everything I used to do has gone, I used to be up hear as head of IT running a £5m budget, now I can’t even do the budget in my own house” [P1, TIA] |
| - Mood (anger, mood swings, frustration) | “This is going to sound a bit wrong but my anger level was higher. I’m a very passive person anyway, so that’s probably more noticeable in me. I think because of the fatigue, I would get more irritated quicker. That irritation results in responses to that, if you like.” [P3, minor stroke]  “But really I mean anger, I’ve got a lot of anger, only Sunday you’d have thought world war three had started in this house between me and the wife and it’s just the smallest thing and I just blew, it’s unlike me because that ain’t me. But mood swings I find a hell of a lot of mood swings lately.” [P1, TIA] |
| - Emotionalism | “I’m acknowledging that I feel emotional, I’m acknowledging the reason that I feel it, I’m acknowledging that I cry easily…” [P7, TIA] |
| **Cognitive** |  |
| - Executive functioning | “…he realised he wouldn’t be able to go home to build that wardrobe cause he wouldn’t be able to follow the instructions which was a big loss for him. And just that idea who he thought he always was, is no longer there, it’s a different guy there.” [H15, psychologist]  “It’s like I used to cook the meals and something like spaghetti bolognaise, simplest thing probably ever, meh, it’s strange I can’t put things in order, miss things out. Feel a bit of an idiot trying to find, I’ll write it down this time what I should be doing, what should I be doing, it’s planning, planning a journey. And I say I know I’m going to the doctors, bloody Tesco why am I at Tesco, I know I’m going to the doctors.” [P1, TIA] |
| - Memory | “…they’re [memory problems] marginal; nothing that anybody apart from my wife would pick up on.” [P3, minor stroke]  “… I need to write much more things down, I'm still doing things forgetting that I've done it or forgetting that I haven't done it…” [P9, minor stroke] |
| - Attention | “…the other thing that he was suffering from, which was a little bit harder to kind of sort out was he felt that he had quite a lack of, a difficulty in concentration and difficulty in focussing and that really was his main worry about returning to work…” [H8, GP] |
| - Language | “…because with my speech comes my confidence because I haven’t go the speech anymore my confidence has gone right back down to basics” [P10, minor stroke] |
| **Fatigue** | "[fatigue] that's the other biggest impact because I literally, I can't do too much…” [P7, TIA]  "And like, its very tired as well, its like, I just feel, oh, take me away from here, I cannot be here or I got in and I try to look for places and go in for five minutes and then I'll say, no we need to leave, I can't do it" [P9, minor stroke]  “I know the tiredness I think was and I’m still, this is 12 weeks and I’m back at work full time and I’m still amazed, I’m fine all day but I’m still amazed what time I’m having to go to bed just to keep up and I’ll notice at the end of the day, like normally I’m the one that does the percentages in the office, yesterday I was sitting going, I don’t even know what I’m doing, you know, by 5 o’clock and I’m not normally like that…” [P8, TIA]  “…so probably two weeks of that if I got out the shower and dried my hair I had to go back to sleep, I was absolutely shattered…” [P8, TIA] |
| **Physical problems** |  |
| - Minor weakness | “And then a couple of times I crashed the gears and I realised that I wasn’t giving my left foot enough welly, so now when I change the gear, I concentrate on my foot. And that has solved that problem” [P11, minor stroke]  “They’ll come to clinic and give that list and say, ‘I don’t feel I can go back to work yet. I’m still too tired’ or ‘My arm is a bit weak but everyone said it was minor and it would go away’.” [H3, physiotherapist] |
| - Altered sensation | “I do get this sensation every so often in my feet where it feels like I’m walking on ice. It doesn’t matter what the surface is. It just feels like I’m going to slip.” [P2, minor stroke] |
| - Pain | “I have pain, so I have pain in my face and my ear and my hand … when there’s too much input, it get too much in my head and then the pain comes.” [P9, minor stroke] |
| - Speech | “Because sometimes if I get stressed ... my speech starts to go, it’s frustrating because I think they’re watching me with this thing rather than just watching me... I feel it inside when I talk, I know sometimes I’m slurring and I sound like a bit of a fool...” [P1, TIA] |
| - Headaches | “Headache and sort of headache frequency increasing post TIA is quite common.” [H22, stroke nurse] |
| - Swallowing problems | “I have a problem with swallowing on the right side of my throat, I was meant to have a swallow test or something, that never happened which is not unusual for the hospital to say oh you’ll have to have a swallow test, then nothing happens. But yes I have trouble swallowing , food gets stuck, it’s strange it’s always the right hand side, nothing on the left, nothing, just the right hand side yeah.” [P1, TIA] |
| **No residual problems** | “I mean once it had passed and all the feeling came back, you forget about it... it’s like you break your leg, once you get better you forget about it don’t you?” [P4, TIA] |

**eTable 4: Impact of TIA and minor stroke on patients’ lives: subthemes and example quotes.**

| **Subtheme** | **Quote** |
| --- | --- |
| **Return to work/ education** | “I struggled in the job being the job it was, just couldn’t get the, even simple things I’d done for many years, many years I’ve done this kind of work.” [P1, TIA]  “I need to take more breaks, I cannot sit in two, three hour meetings, that kills me so I need to have more breaks and I need to write much more things down, I’m still doing things forgetting that I’ve done it or forgetting that I haven’t done it, I put in the same meeting twice with the team… I’m almost up to full time … but not full capacity because I notice that I’m not this quick or smart as…, I need to focus much more.” [P9, minor stroke]  “Yeah I’m having to record everything now at college, I will write it down because I’m like, what did he say and then I have to go home and put my recorder on or get my books out and go, ah that was it. My mock, I did a mock exam the other day, six questions he give us the time and I was like, I ain’t finished it, two years ago I would have done it like that…” [P10, minor stroke]  “At the moment I’m still on light duties… I don’t want to use the machinery just yet.” [P12, minor stroke]  “I was going to be a senior social worker and I gave up that opportunity because I’d just been diagnosed, I’d had the interview, so I hadn’t got the confidence then. So, I think it knocked what could happen in the future” [P2, TIA]  “…depending on their workload, you know, it depends on the type of job, if it’s a more manual job, then potentially they’re ok. If it’s much more cognitively demanding, they perhaps struggle with that a little bit more in terms of their executive functioning.” [H14, OT] |
| **Relationships with family/ friends** | “Mood swings are the worst for my wife, she suffers the most… so I try to watch what I say, most times it’s quietness between us because I’m scared to say something that will just trigger one of these [mood swing] it’s a stressful time for her…” [P1, TIA]  “But I tend to shy away, even my own parents, every Sunday I go to my parent’s house where I’ll sit at the back, it’s hard for me to join in the conversation now, it’s hard for me to take in what people say to me.” [P1, TIA]  “I mean my son is probably the most important thing. And its having to have to tell him to you know, be less loud and I can’t manage when he gets too intense or if he’s invited to birthday party and they do that with a lot of children, I struggle, I hate this… so that feels sad that he has to adjust.” [P9, minor stroke]  “My daughter said to me that her biggest fear… was that I wouldn’t be able to talk to her anymore and we have fantastic, we have a really close relationship and lots of conversations and she said she was terrified on that day that I would never be able to speak to her again…” [P7, TIA]  “…but surprising things that people did that just really, you know, one of my sisters, who I’d had a difficult relationship with, she packed up her things and came up the next day so since then she’s messaged me every day, she’s totally been there…” [P7, TIA] |
| **Social lives and daily living** | “That’s why socially I keep away from it because I ain’t got to go through what I went through with my mates” [P1, TIA].  “My quality of life is affected because I can’t go back to doing things that I like to do, I haven’t got… I’m not working at the moment, I do piano lessons*,* I’m not doing piano lessons, I’m not going to the gym to the same degree, my personal trainer basically dropped me because I couldn’t really do very much so you know it’s like all of that that you have lost…” [P7, TIA]  “We have all sorts of interesting conversations about people’s personal lives as well, can they fly, can they go in a hot tub, can they have sex … and holidays and looking after children. You would be amazed at what comes out in our conversations that probably doesn’t with a consultant.” [H22, nurse] |
| **Acceptance** | “I was totally in shock, I knew what had happened but I couldn’t believe what had happened” [P8, TIA]  "I was too shocked to ask the questions and there was nobody with me because I was convinced I hadn’t had one." [P2, TIA]  “what have I done, why have I got this, you know, what have I done wrong, there was all this kind of feeling that I’d done something terrible, wrong, that I’d brought this on myself” [P8, TIA]. |
| **Hidden impairments** | “I know it’s there but people who look at me don’t see it. They say oh you look okay. That’s the worst thing is when you say, oh you look okay, I might look good, I don’t feel it you know” [P1, TIA] |
| **Self-loss** | “for me is quite a big loss really because that [exercise] was quite a big part of what I did” [P8, TIA]  “[referring to being unable to exercise] …you know, but for me that was a real biggie because it was like my whole life was gone in a second, you know…” [P7, TIA] |

**eTable 5: Experience of follow-up care and sources of support post-TIA and minor stroke: subthemes and example quotes.**

| **Subtheme** | **Quote** |
| --- | --- |
| **Experience of follow-up care** | “… there was this big gap in the four weeks, it’s the crucial four weeks after I think when you just, that’s when the anxiety would get hold I think if it was going to because it would start building and you are just left and then following that they just sort of go, oh no you are fine…” [P8, TIA]  “I just didn’t feel very listened to either because it was a TIA, because I’m healthy, because there was no permanent… so all the things I’m lucky for really, but they made me very easy to just dismiss I think as well I was cross about that if I’m honest…” [P8, TIA]  “I’m sure some of my colleagues don’t [follow-up patients] and some probably follow-up more than I do. We do have a, for follow-up we do have a nurse lead follow-up clinic. Which I have access to, but I don’t use a lot. And again, there’s some variation in practice amongst the five stroke physicians about how much they use that clinic.” [H20, consultant] |
| **Information needs** | “But I like to see them just because dare I say, in the acute phase when patients are seen an awful lot of information ... and so the amount of information that they absorb is tricky, but also I’m not sure whether there is necessarily all the information that’s given to them at the time. Maybe it is, maybe they forget about it, maybe they get given patient information leaflets, but it’s just confirming the understanding moving forward that I like to do.” [H11, GP]  “I can explain that and feel that I can get it across in an understandable way for the patient without using, I think terminology is important, you know, I’ve been on the ward and I’ve heard doctors tell them [patients] what’s gone on, well if you’re not from a medical background you wouldn’t have a clue, so I think yeah, terminology is really important.” [H16, AHP]  “My biggest criticism would be that the hospital advice is very generalised, there was nothing based on me, on the person that I was at that time…” [P7, TIA]  “So when I typed in mini stroke and all this information came up I was like, well which one is the best one for me. and then you read this one thing and you read something else and it’s different to that one but it [contradicts], that’s it and I’m like hang on a minute that one’s just said that one, that one’s saying that one, I’m like well which one is it now. So I think there’s too much information out there because it can be really confusing.” [P10, minor stroke] |
| **Stroke prevention** | “Rightly or wrongly I think we have to really make the assumption that the patient has been counselled adequately about that medication and why they’re being put on it… it wouldn’t be feasible for every specialist letter we get for strokes and everything else to contact the patient to sort of go through the, we wouldn’t do anything else really. So we add the medication to the repeat prescription…” [GP, H13]  “A lot of the time it’s about medication and sort of having a look at their new medications that they’ve been put on. Because unfortunately sometimes in hospital … you get this kind of ‘protocolised’ ‘right, you need to start Aspirin, right you need to start Clopidogrel’ … then they come to see you and you think ‘well, actually you can’t take that’.” [H11, GP]  “So, if they have an abnormal test, such as abnormal heart rate recording that shows there’s atrial fibrillation and they tend need an anticoagulation which is a radicle sort of change in their medication and has to happen quickly, I don’t leave that for the GP and none of my colleagues.” [H17, consultant]  “And again, I’m generally a bit reluctant to treat blood pressure cause I’m a Neurologist, So, neurologist probably lack a bit of expertise on treating blood pressure.” [H20, consultant]  “So, we talk about stopping smoking and healthy diet and exercise but it’s a fairly brief discussion and don’t really feel I have time in the clinic to do that in great depth.” [consultant, H20] |
| **Holistic needs** | “Obviously being a TIA they will have all made a full recovery…” [H10, GP]  “I don't think I will bring back somebody to manage their mood and fatigue because I don’t feel competent in doing that and probably I'm not.” [H17, consultant]  “I: And did you mention to them things about your confidence and your speech, those kind of things, did you discuss it in those appointments?  IV: No because that was just for like my bloods and my temperatures and my weight, just basic, no …” [P10, minor stroke]  “Three, four, five or six months afterwards, I recognised that I needed some form of psychological treatment which is why I went to see a psychologist. That was done through the doctor which was very good, no doubt. I think trying to get that was difficult… I had to press quite hard to get a psychologist review, if you like. Once that ball was rolling, you fall into process almost but it’s that initial feeling, ‘I feel like I need this. You, as a medical community, don’t recognise that.’ Therefore, they don’t think you need it.” [P3, minor stroke]  “And my mum stayed here, so she helped me at home, my son was four at that time and I was sleeping a lot, I was very, very tired and four year old is quite intense piece of kid, so what happened was that she was, she stayed with us…” [P9, minor stroke]  “I don’t feel I should bother them, it’s strange you know TIA, it ain’t stroke… I just don’t feel that I’m at that level to say yeah, yeah come and help me…” [P1, TIA]  “Generally, no, we don’t have the time [to talk about lifestyle], you know to go through that… so we give the stroke association leaflet so that contains all the information regarding that. So, we ask the patients to read that and to make the necessary adjustment.” [H7, consultant]  “But she [doctor] told me things to do, like word searches, relaxing jigsaw puzzles, to keep my mind active…” [P11, minor stroke] |
